# Validation of Gene Profiles for Analysis of Regional Lymphatic Metastases in Head and Neck Squamous Cell Carcinoma

**DOI:** 10.3389/fmolb.2020.00003

**Published:** 2020-02-04

**Authors:** Zhenrong Hu, Ranran Yang, Li Li, Lu Mao, Shuli Liu, Shichong Qiao, Guoxin Ren, Jingzhou Hu

**Affiliations:** ^1^School of Stomatology, Weifang Medical University, Weifang, China; ^2^Department of Oral Maxillofacial-Head and Neck Oncology, Shanghai Ninth People's Hospital, Shanghai Jiao Tong University School of Medicine, Shanghai, China; ^3^Shanghai Key Laboratory of Stomatology & Shanghai Research Institute of Stomatology, Shanghai, China; ^4^National Clinical Research Center of Stomatology, Shanghai, China; ^5^Shanghai Key Laboratory of Stomatology, Department of Oral and Maxillo-facial Implantology, School of Medicine, Shanghai Ninth People's Hospital, Shanghai Jiao Tong University, Shanghai, China

**Keywords:** head and neck squamous cell carcinoma (HNSCC), lymph nodes, metastasis, mRNA expression, CSCs and immune suppression markers

## Abstract

The progress of Head and Neck Squamous Cell Carcinoma (HNSCC) is dependent on both cancer stem cells (CSCs) and immune suppression. This study was designed to evaluate the distribution of CSCs and the characteristic immune suppression status in HNSCC primary tumors and lymph nodes. A total of 303 lymph nodes from 25 patients, as well as tumor and adjacent normal tissue samples, were evaluated by a quantitative PCR assay of the markers of CSCs and the characteristic immune suppression. Expressions of selected genes in The Cancer Genome Atlas (TCGA) datasets were also analyzed. In the primary tumors, we found that expressions of CSCs markers *(ALDH1L1, PECAM1, PROM1)* were down-regulated, while immune suppression *markers FOXP3, CD47, EGFR, SOX2*, and *TGFB1* were up-regulated significantly when compared to that in adjacent normal tissues. In the lymph nodes, expressions of both CSCs, and immune suppression *markers* were upregulated significantly compared with that in primary tumors. The mRNA expression of selected CSCs and immune suppression markers exhibited the highest expression in the level II of metastasis, then declined in the level III and remained constant at a reduced value in levels IV and V of metastases. These results reveal a comprehensive understanding of the unique genetic characteristics associated with metastatic loci and potential routes of lymphatic dissemination of HNSCC, which helps to explain why the level II has a high incidence of lymph node metastasis, and why skip metastasis straight to the level IV or level V is rarely found in the clinic.

## Introduction

Head and Neck squamous cell carcinoma (HNSCC) is ranked as the sixth most common cancer in the world, with almost 600,000 new cases occurring every year (Bray et al., 2018). More than 50% of HNSCC patients present with metastasis to local lymph nodes at the time of diagnosis. Regional lymph nodes metastasis not only indicates poor survival, but is also a major prognostic factor for the determination of the appropriate treatment (Ozdek et al., 2000; Michikawa et al., 2012). Patients with regional lymph nodes metastasis present a 30–60% 5-year survival rate compared with ~85% for patients without synchronous nodal dissemination (de Juan et al., 2013). Furthermore, regional or metastatic recurrence are more likely to arise in the patients with synchronous nodal dissemination after completing synthetic serial therapy (Wan et al., 2012). However, such regional or metastatic recurrence in HNSCC is generally considered to be incurable and resistant to conventional treatment, with almost 22 months median survival rate in patients receiving salvage surgery or irradiation, and ~12 months for those undergoing palliative chemotherapy alone (Leon et al., 2012; Ho et al., 2014).

Increasing evidence demonstrates a complex, nonlinear, branched evolution model of subclonal populations in cancers (Ginos et al., 2004). The model characterized as a dynamic process that minor subclones likely expand under the selective pressure of therapy (Ginos et al., 2004). It has been reported that hematological malignancies reveal a distinct pattern of clonal evolution in the development of therapeutic resistance and relapse (Tomasson et al., 2012). A microarray-based study has identified a number of HNSCC metastasis and recurrence associated genes in the tissue of primary or recurrent tumors, unmatched normal mucosa and lymph node metastases, but the clinical implication of these observations remains unknown (Lacko et al., 2012). Another study revealed that the mRNA expression of HNSCC in primary tumors are similar to their respective matched metastatic lymph nodes (Reis et al., 2011). These studies have provided a great insight into the genetic alterations underlying the process of metastasis in HNSCC, which will help us to identify novel therapeutic targets.

The extent of lymph node metastasis is an important prognostic factor for locally advanced HNSCC, and previous studies have reported that skip metastasis to inferior cervical lymph nodes at levels III or IV in the absence of demonstrable involvement of levels I and II is rarely found in HNSCC (Amin et al., 2017). However, the genetic alterations and underlying mechanism in the process of nodal dissemination are poorly understood and little is known about the impact of the level of lymph nodes metastasis (LNM) for patients with HNSCC. It presents a great challenge to develop more effective therapeutic strategies to prevent metastases and recurrence. For the purpose of uncovering the CSCs and immune suppression-related genetic alterations underlying metastasis in HNSCC, we chose 15 of the CSCs and immune suppression-related genes that have been reported in the primary expression of HNSCC before (Kosan and Kunz, 2002; Grosse-Gehling et al., 2013; Nor et al., 2014; Prakasam et al., 2014; Yang et al., 2014; Hartomo et al., 2015; Wu et al., 2015; Ji, 2016; Ren et al., 2016) performed RT-PCR to examine the expression levels of in cancer tissues, lymph nodes, and the matched normal tissues from the same patients with synchronous nodal metastases.

## Materials and Methods

### Cohorts of Enrolled HNSCC Patients

Twenty-five patients diagnosed with HNSCC and subjected to primary operation in Oral and Maxillofacial–Head and Neck Oncology Department of Shanghai Ninth People's Hospital between 2015 and 2016 were screened for the experiments. All patients recruited had not had chemotherapy or radiotherapy prior to the surgical treatment and the patients who underwent neo-adjuvant chemo or radiotherapy were excluded from our study. The mean age of 25 participants (17 men and 8 women) was 57, ranging from 52 to 74. The samples were collected during the surgery and immediately frozen, including cancer tissues, adjacent normal tissues and lymph nodes from the same patient. Adjacent normal tissues were required to be more than 2 cm from the tumor margins in the same patient. The categorization of neck dissection samples was in accordance with the topographic classification of cervical lymph node levels suggested by Gregoire et al. (2014) as IA, IB, IIA, IIB, III, IV, V. Metastatic lymph nodes were confirmed by hematoxylin-eosin (HE) staining and observed by two pathologists independently. The written informed consent of all patients was obtained, following the protocols approved by Shanghai Ninth People's Hospital Ethical Committee and the study was performed in accordance with the Declaration of Helsinki.

### Exclusion Criteria

The patients in one of the following situations were excluded in this study (Zhi et al., 2015).

Patients with local recurrences or second primary tumors.

Patients who were HPV positive.

Patients who experienced chemo- or radiotherapy prior to this study.

### The Cancer Genome Atlas (TCGA) Datasets Analysis

The selected gene expression was downloaded from mRNA expression detection platform RNA-seq version 2 (level 3) in TCGA data portal (http://cancergenome.nih.gov/). The final number of HNSCC patients included was 509. The statistical programing software R (version 2.14.1) was used to analyze the datasets with the statistical significance at *P* < 0.05. The normalized counts (cancer and adjacent normal) were used to compare the gene expression (RNA-Seq version 2).

### mRNA Expression Profiling

Trizol reagent (Life Technologies, USA) was used to extract the total RNAs of all the acquired samples, and iScriptTMcDNA synthesis kit (Bio-Rad, CA) was used to reverse transcription (RT). The FastSYBR Green master mix with Rox (Life Technologies, USA) was used to perform the quantitative PCR. Fifteen genes were evaluated by quantitative RT-PCR. The primers for *IRF1, IFNAR2, FOXP3, TMEM173, CD47, PECAM1, BMI-1, TWISTNB, ALDH1L1, PROM1, EGFR, SOX2, TGFB1, SMAD3*, and *STAT3* were purchased from SABiosciences. The primer sequences of the genes are listed in Table S1. The gene β-actin was chosen to be the control gene for normalization.

### Statistical Analysis

Data from more than three independent experiments are represented as the mean ± standard deviation (SD). Pared t test was used to do statistical analyses comparing the genes expression between primary and adjacent normal tissues of HNSCC patients, Mann Whitney test was performed to analyze the genes expression in metastatic nodes and non-metastatic nodes and data from TCGA, Kruskal–Wallis test was used to access the expression of metastatic nodes in different levels. Mean-normalized mRNA expression value in non-metastasis lymph nodes were chosen to be control for the entire cohort of lymph nodes. The relative value of each gene was calculated as follow: The ΔΔCt = tumor ΔCt-control ΔCt, fold change of mRNA was obtained as 2^(−ΔΔCt)^. We considered the data was significant at *P* < 0.05.

## Results

### Clinicopathologic Characteristics of HNSCC Patient Samples

Clinicopathologic characteristics for the study groups (*n* = 25) including age, gender, tobacco and alcohol history, tumor site, and AJCC stage [which was according to AJCC 8th Edition (Tao et al., 2006)] are summarized in Table 1. Patients' ages ranged from 52 to 74 with a mean age of 57 ± 7.8 years. The gender distribution was 68% male (17/25) and 32% female (8/25). Forty eight percent (12/25) had a history of substantial tobacco exposure (generally >20 pack years), and 44% (11/25) had documented alcohol use. The lesion sites involved were oropharynx (24%) and oral cavity (76%). 4, 36, 24, and 36% of the patients had T1, T2, T3, and T4 tumors, respectively, while 4, 20, 28, and 48% were staged to I, II, III, and IV, accordingly. The majority of the patients had subsequent therapy with radiation (92%) after the surgery. Of all the 25 patients, 13 patients were diagnosed with synchronous nodal metastasis. Metastatic carcinomatous cells were observed in 27 (2.5%) of the 303 lymph nodes. However, there were only 2 cases of skip metastases to level IV and 1 case of skip metastasis for level V. The details of these results are presented in Table 2.

**Table 1:** Clinicopathological parameters of enrolled HNSCC patients.

**No**.	**Tobacco**	**Alcohol**	**Site of tumor origin**	**Pathological stage**	**AJCC stage**
1	YES	YES	Tongue (oro-pharyngeal)	pT4aN0M0	IVa
2	YES	YES	Mouth floor	pT2N0M0	II
3	NO	NO	Buccal	pT4aN0M0	IVa
4	NO	NO	Tongue	pT2N1M0	III
5	YES	YES	Mouth floor	pT2N0M0	II
6	YES	YES	Gingiva	pT2N2aMO	IVa
7	NO	NO	Gingiva	pT2N2bM0	IVa
8	YES	YES	Mouth floor	pT3N1MO	III
9	YES	NO	Tongue	pT3N0M0	III
10	NO	NO	Palate	pT4N0M0	IVa
11	YES	YES	Mouth floor	pT3N1MO	III
12	YES	YES	Mouth floor	pT4aN2cM0	IVa
13	NO	NO	Tongue	pT3N1M0	III
14	YES	YES	Tongue (oro-pharyngeal)	pT4N0M0	IVa
15	NO	NO	Tongue	pT3N1M0	III
16	NO	NO	Tongue (oro-pharyngeal)	pT4N1M0	IVa
17	YES	YES	Mouth floor	pT4N2bM0	IVa
18	NO	YES	Gingiva	pT2N2bM0	IVa
19	NO	NO	Tongue	pT2N0M0	II
20	YES	YES	Tongue	pT1N0M0	I
21	NO	NO	Tongue (oro-pharyngeal)	pT3N1M0	III
22	YES	NO	Tongue (oro-pharyngeal)	pT4N0M0	IVa
23	NO	NO	Tongue (oro-pharyngeal)	pT4N1M0	IVa
24	NO	NO	Buccal	pT2N0M0	II
25	NO	NO	Buccal	pT2N0M0	II

**Table 2:** Results of histopathologic examination of various cervical lymph node levels.

	**Lymph node levels**							
**Numbers**	**Level IA**	**Level IB**	**Level IIA**	**Level IIB**	**Level III**	**Level IV**	**Level V**	**Total**
Metastasis	4	10	3	2	5	2	1	27
Non-metastasis	39	40	25	38	62	52	20	276
Total	43	50	28	40	67	54	21	303

### Gene Expression Profiles in HNSCC TCGA Dataset

**Figure 1:**
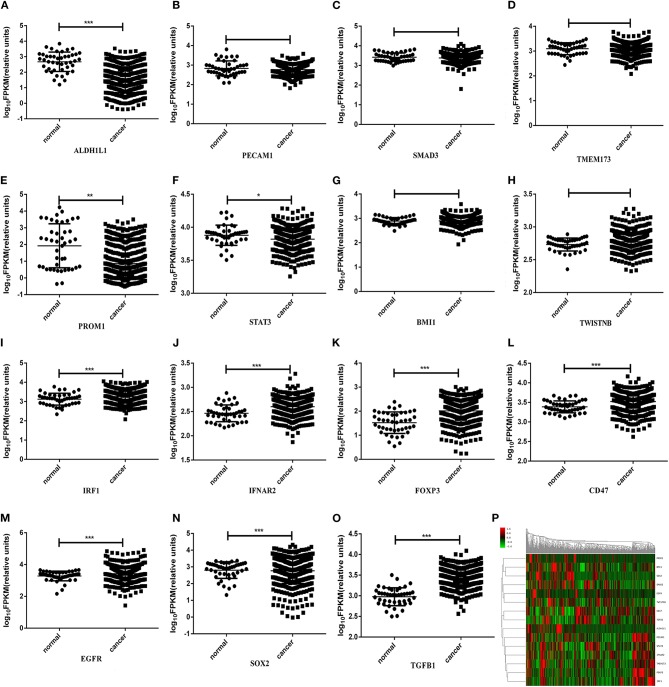
FPKM values of *ALDH1L1* **(A)**, *PECAM1* **(B)**, *SMAD3* **(C)**, *TMEM173* **(D)**, *PROM1* **(E)**, *STAT3* **(F)**, *BMI-1* **(G)**, *TWISTNB* **(H)**, *IRF1* **(I)**, *IFNAR2* **(J)**, *FOXP3* **(K)**, *CD47* **(L)**, *EGFR* **(M)**, *SOX2* **(N)**, and *TGF*β*1* **(O)** expression of HNSCC patients in TCGA datasets; **(P)** Heatmap of gene expression profiles of HNSCC patients in TCGA datasets. Gene expression values are calculated based on the log transform of FPKM values determined from RNA sequencing analyses. High expression is depicted by red; low expression is depicted by green, normalized by row z scores (legend). *P < 0.05, **P < 0.01, ***P < 0.001.

Since differentially expressed genes of the transcriptome data of HNSCC in TCGA dataset have been reported (Cancer Genome Atlas, 2015; Yan et al., 2016), we further examined the expression profiles of the selected genes (i.e., *IRF1, IFNAR2, FOXP3, TMEM173, CD47, PECAM1, BMI-1, TWISTNB, ALDH1L1, PROM1, EGFR, SOX2, TGFB1, SMAD3*, and *STAT3*) in the primary tumors of HNSCC. The Clinicopathologic characteristics of TCGA dataset was shown in Table 3. It enrolled 522 HNSCC patients and lesion sites involved 312 in oral cavity (59.8%), 115 in Larynx (22.0%), 82 in oropharynx (15.7%), 10 in hypopharynx (1.9%), and 3 in Lip (0.6%). Forty four of them were diagnosed with HPV infection. This is shown in Figure 1, in which *ALDH1L1, PECAM1, SMAD3, TMEM173, PROM1*, and *STAT3* were expressed lower, and the rest of the genes were expressed higher in primary tumors than in adjacent normal tissues. Among them, the expression levels of *IRF1, IFNAR2, FOXP3, CD47, ALDH1L1, PROM1, EGFR, SOX2, TGFB1*, and *STAT3* were significant (*P* < 0.05) between tumors and normal tissues (Figure 1).

**Table 3:** Clinicopathological parameters of HNSCC patients in TCGA dataset.

**Characteristic**	**Number**	**Characteristic**	**Number**
Gender		Age	
Male	381	<60	288
Female	141	≥ 60	233
		Not available	1
Smoking status		Alcohol	
Smoker	388	Yes	351
Nonsmoker	121	No	162
Not available	13	Not available	9
Tumor stage		Lymph node stage	
I-II	186	N0-1	327
III-IV	320	N2-3	173
Tx	12	Nx	18
Not available	4	Not available	4
Metastasis stage		AJCC stage	
M0	492	I-II	120
M1	4	III-IV	388
Mx	21	Not available	14
Not available	5		
Primary region		Race	
Gingiva	18	American Indian	2
Tongue	131	Asian	11
Hard palate	7	Black American	45
Floor of mouth	61	White American	447
Bucca	22	Not Available	17
Tonsil	45	HPV	
oropharynx	37	Positive	44
hypopharynx	10	Negative	81
Larynx	115	Not Available	397
Lip	3		
Oral Cavity	73		

### Examination of mRNA Expression in Primary Tumors by RT-PCR Detection

**Figure 2:**
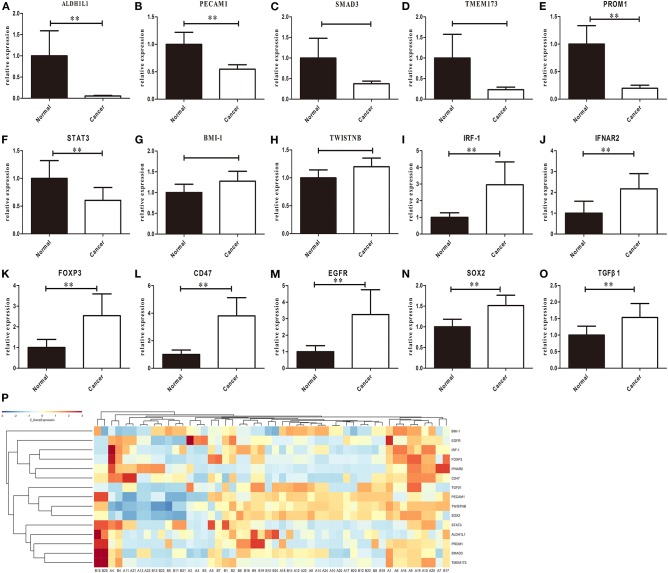
RT-PCR results of *ALDH1L1* **(A)**, *PECAM1* **(B)**, *SMAD3* **(C)**, *TMEM173* **(D)**, *PROM1* **(E)**, *STAT3* **(F)**, *BMI-1* **(G)**, *TWISTNB* **(H)**, *IRF1* **(I)**, *IFNAR2* **(J)**, *FOXP3* **(K)**, *CD47* **(L)**, *EGFR* **(M)**, *SOX2* **(N)**, and *TGF*β*1* **(O)** expression in primary tumors. Each bar represents the average log2 gene mRNA expression level of the paired tumor and adjacent normal tissues (*n* = 25 per group). Eleven genes showed significant changes between paired tumor and adjacent normal tissues **(P)** Heatmap of gene expression profiles of HNSCC patients in the primary of HNSCC, Gene expression values are calculated based on the log transform of FPKM values determined from RNA sequencing analyses. High expression is depicted by red; low expression is depicted by green, normalized by row z scores (legend) (**p < 0.05, *t*-test).

**Figure 3:**
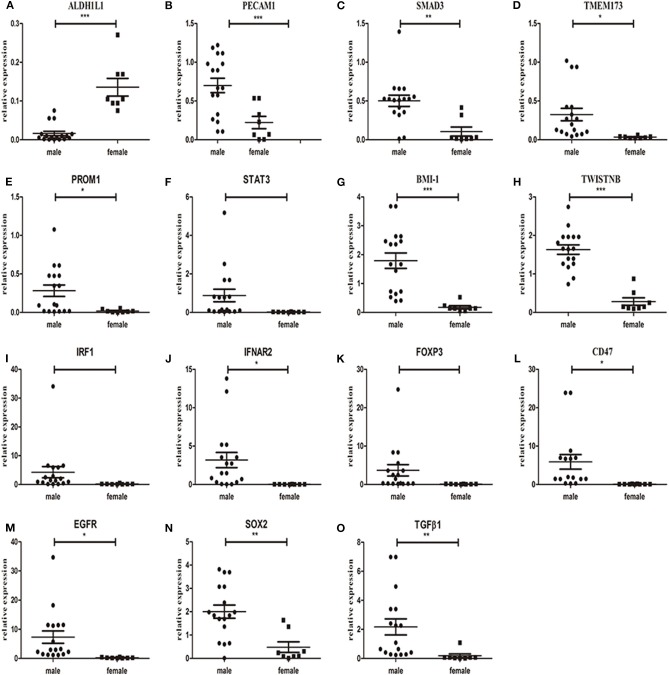
mRNA expression of *ALDH1L1* **(A)**, *PECAM1* **(B)**, *SMAD3* **(C)**, *TMEM173* **(D)**, *PROM1* **(E)**, *STAT3* **(F)**, *BMI-1* **(G)**, *TWISTNB* **(H)**, *IRF1* **(I)**, *IFNAR2* **(J)**, *FOXP3* **(K)**, *CD47* **(L)**, *EGFR* **(M)**, *SOX2* **(N)**, and *TGF*β*1* **(O)** in the primary of tumor samples between male and female patients. *P < 0.05, **P < 0.01, ***P < 0.001.

To further investigate the above-mentioned gene expression in our HNSCC samples, we validated the mRNA expression using real time RT-PCR detection. The differential levels of mRNA expression (–ΔΔCt) of the 15 genes in each of the 25 individuals HNSCC sample are shown in Figure 2. Except for *PECAM1*, which showed a significant decrease (*P* < 0.05), while the TCGA dataset revealed no significant difference, the expression levels (up or down) of the rest 14 selected genes were consistent with TCGA profiles as validated by real time RT-PCR assay for all recruited patients and a clustering analysis was performed in the primary of HNSCC (Figure 2). Furthermore, there were no significant differences between men and women in normal tissue, but it showed gender difference with male dominancy in the primary (Figure 3 and Figure S1). We also found there were no significant differences between sex and risk factors (such as smoking or drinking) (*p* = 0.896 and 0.694, respectively). It suggested that sex factor might play a role in the formation of CSCs and immune suppression and might be more susceptible to metastasis.

### Gene Expression in Different Lymph Nodes

**Figure 4:**
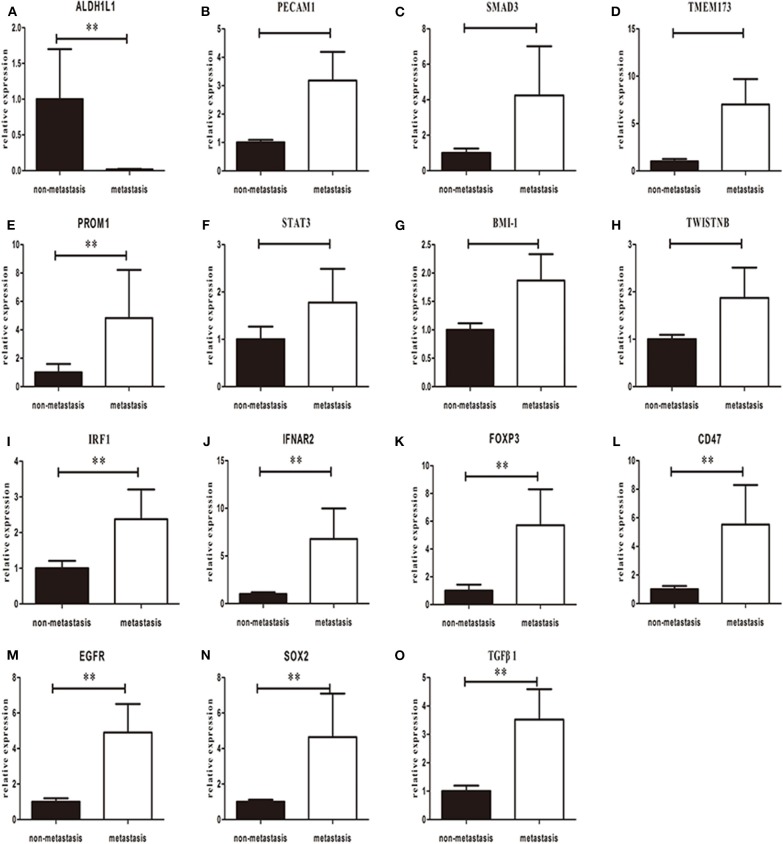
Overall mean-normalized mRNA expression of *ALDH1L1* **(A)**, *PECAM1* **(B)**, *SMAD3* **(C)**, *TMEM173* **(D)**, *PROM1* **(E)**, *STAT3* **(F)**, *BMI-1* **(G)**, *TWISTNB* **(H)**, *IRF1* **(I)**, *IFNAR2* **(J)**, *FOXP3* **(K)**, *CD47* **(L)**, *EGFR* **(M)**, *SOX2* **(N)**, and *TGF*β*1* **(O)** in lymph nodes. Each bar represents the average log2 gene mRNA expression level of the non-metastatic and metastatic lymph nodes. *P*-values are calculated by Wilcoxon rank-sum test. (**p < 0.05).

**Figure 5:**
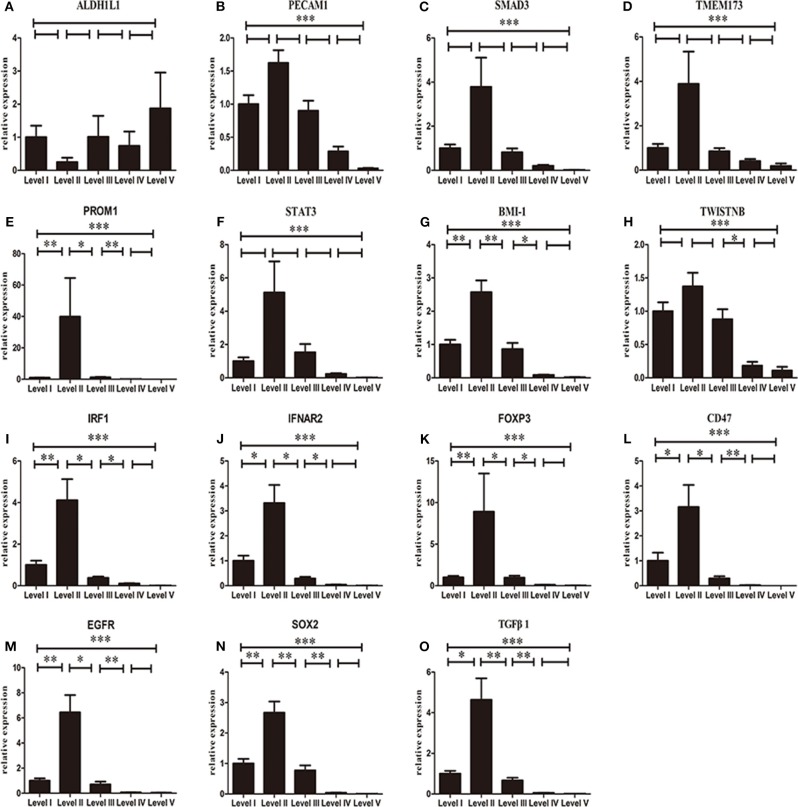
Normalized 2 ^(−ΔΔCt)^ mRNA expression of *ALDH1L1* **(A)**, *PECAM1* **(B)**, *SMAD3* **(C)**, *TMEM173* **(D)**, *PROM1* **(E)**, *STAT3* **(F)**, *BMI-1* **(G)**, *TWISTNB* **(H)**, *IRF1* **(I)**, *IFNAR2* **(J)**, *FOXP3* **(K)**, *CD47* **(L)**, *EGFR* **(M)**, *SOX2* **(N)**, and *TGF*β*1* **(O)** in different levels of lymph nodes. Each bar represents the average log2 gene mRNA expression level of the metastatic lymph nodes in each individual level. *P*-values are calculated by Kruskal–Wallis test (*p < 0.05, **p < 0.01, ***p < 0.001).

We further compared the mRNA expression of the selected genes between metastatic nodes and non-metastatic nodes in cervical lymph nodes using RT-PCR assay. Similar to the expression levels in primary tumors, the expressions of *CD47, EGFR, FOXP3, IFNAR2, IRF1, SOX2, BMI-1, PROM1*, and *TGFB1* were upregulated significantly in metastatic nodes when compared to non-metastatic nodes (Figure 4). *STAT3* and *PECAM1* were decreased in primary but upregulated in lymph nodes (*P* > 0.05) (Figures 2, 3). One exception is *ALDH1L1* that exhibited a low mRNA expression level in metastatic nodes (Figure 3). Finally, we analyzed the mRNA expression of the selected genes among different levels of metastatic lymph nodes. We found that all the selected genes except *ALDH1L1* followed a similar change from level I–level V metastatic nodes, in which all the genes reached the highest expression in level II, declined in level III and remained at a constant low value in level IV and level V of metastatic nodes (Figure 5). Also, it revealed gender difference with male dominancy in the lymphatic loci of HNSCC (Figure S2).

## Discussion

The nodal dissemination of carcinomatous cell is a vital determinant of prognosis in patients with HNSCC, as the overall survival rate of patients with metastatic lymph nodes decreased by a half compared with that without nodal implicated (Yan et al., 2014). Keeping track of tumor cells through lymphatic metastasis and microenvironment in lymph nodes is particularly vital for treatment (Owens et al., 2014). As far as we know, there were no studies that investigated the mRNA expression in an individual cervical lymph node level. Hence, the TCGA database was used in our study to perform the unbiased, large-scale analysis of 15 metastasis and tumor microenvironment associated genes. The results showed a significant change of related gene expression through both the HNSCC TCGA-based and our tumor-based analyses. Furthermore, the head and neck squamous cancer (HNSCC) with HPV-negative (HPV^−^) always occurred in an older patient population and their clinical outcomes were unquestionably worse than HPV^+^ HNSCC, and few HNSCCs are associated with HPV infection. Therefore, our results provide a model to determine gene expression patterns in both the primary tumors and metastatic lymph nodes of HPV^−^ HNSCC.

*IFNAR2* encodes a protein that forms one of the two chains of a receptor for interferons alpha and beta (Zhang et al., 2015). The protein encoded by IRF1 functions as a transcription activator of interferons α-,β-, and γ-induced transcription (Choe et al., 2015) *TMEM173* encodes a transmembrane protein that functions as a pattern recognition receptor that activates type I interferon responses (Schreiber and Piehler, 2015; West et al., 2015). All three genes promote the IFN-signaling in the lymph node of HNSCC (Li and Flavell, 2008). TGF-β1 encodes a secreted ligand of the transforming growth factor-beta superfamily proteins (Kudinov et al., 2016). The band of these ligands and various TGF-β receptors result in the recruitment and stimulation of *SMAD3* that promotes the process of carcinogenesis (Chaturvedi et al., 2014; Wang et al., 2016). IFN-α, IFN-β and TGF-β play important roles in regulating the activity of lymph node stromal cells embracing lymphatic endothelial cells (LECs), follicular dendritic cells (DCs), and fibroblastic reticular cells (FRCs) (Yang et al., 2014; Ji, 2016). The functional stromal cells may reconstruct and remodel the lymph node, which would produce a unique microenvironment benefit for cancer metastasis (Hartomo et al., 2015). Our RT-PCR results show that are both upregulated in the lymph nodes of HNSCC patients and suggest that these hyperactive lymph node stromal cells may provide a suitable microenvironment for the metastases.

It has been reported that ALDH1 has higher activity in the stem cell subclone of leukemia and some solid tumors (Li et al., 2016). However, *ALDH1L1* showed totally different presentation in distinct types of cancers; mRNA high expressions of *ALDH1L1* were reported to be correlated to higher overall survival rate for breast cancer patients but were revealed as a poor prognostic factor in gastric and prostatic cancers (Prakasam et al., 2014; Wu et al., 2015; Ren et al., 2016). Our result revealed *ALDH1L1* had a lower mRNA expression level in primary and metastatic nodes and exhibited an entirely different behavior compared with other selected genes, indicated that *ALDH1L1* mRNA low expressions is related with metastasis of HNSCC. Our observations showed *SOX2, BMI-1, PROM1* (CD133), and *TWISTNB (TWIST NEIGHBOR)* upregulated in lymph nodes. *SOX2, BMI-1*, and *PROM1* are cancer stem cells-related genes (Kosan and Kunz, 2002; Grosse-Gehling et al., 2013; Nor et al., 2014), and *TWISTNB* is implicated in EMT (Li and Li, 2015). Previous studies indicated that cancer stem cells (CSCs) *in situ* can transform into migrating cancer stem cells (MCSCs) by EMT (Schlereth et al., 2014). Subsequently, the MCSCs disseminate and form metastatic colonies (Li and Li, 2014). Furthermore, direct trans-differentiation of CSCs into LECs occurs during tumor lymphatic metastasis (Chao et al., 2012; Semenza, 2013). As previously mentioned, LECs remodel the lymph nodes that provide a comfortable microenvironment for cancer metastasis. Our observations of gene expression profiles in lymph nodes suggest that CSC may directly convert into LECs and contribute to tumor neovascularization in the metastasis process of HNSCC.

*CD47* is likely involved in the process of evading immunological eradication (Chattopadhyay et al., 2005; Matlung et al., 2017), and FOXP3 is mainly considered as a biomarker of Treg cells that impede the antitumor immune responses in cancer patients (Quante et al., 2013; Triulzi et al., 2013). *STAT3* overexpression in metastatic sites may restrain the immune responses to render an immunosuppressive environment (Punt et al., 2015). PECAM1 (CD31) makes up a large portion of cell intercellular junctions and loss of its function may disrupt cell adhesion (ElShamy et al., 2016). Interaction of hypoxia-surviving cells with the immunosuppressive environment influenced by newly recruited tumor-associated macrophages (TAMs), mesenchymal stromal cells (MSCs), and other types of immune cells most likely form and maintain a necrotic/hypoxic core called “aggressiveness niche” that will be the foster ground for cancer metastasis precursors (Johnson, 2001). In accordance with above reports, our study demonstrates that *CD47* and *FOXP3* were significantly upregulated both in primary site and lymphatic loci, but *STAT3*, and *PECAM1* were only upregulated in lymph nodes. It indicates that there may form a more immunosuppressive environment in the lymph nodes than primary site and may exist an aggressiveness niche to foster metastasis in HNSCC patients.

A previous study revealed that male patients may have higher tendency of cancer-associated gene expression in the primary than matched adjacent normal tissue (Shores et al., 2004). Similarly, the expression of 15 selected genes showed gender differences with male dominancy not only in the primary but also in metastatic loci, with no significant differences between men and women in matched adjacent normal tissue. It indicated that males may be at a higher risk of metastasis than females in HNSCC. The mechanism underlying these expression patterns will be further studied in the future.

It have been reported that a relatively constant and sequential route might exist in the lymphatic drainage of the head and neck region (Ji, 2016). Level I and level II are the most common region of metastases in cancers of the oral cavity, while level III is most likely area of metastases in cancers of hypopharynx (Vishak and Rohan, 2014). Considering the potential mechanisms of the route of lymphatic metastasis remains unknown, our observations of the differential mRNA expressions in respective cervical levels showed that CSC and immunosuppression-related genes achieve a peak value in the level II, but maintain a constant low value in the levels IV and V, which suggests that the level II may mostly provide a necrosis-induced inflammation and hypoxia-induced immunosuppressive environment that seems to be the most fertile ground to generate the tumor cells with metastatic potentials. This supports that carcinomatous cells in the level II tend to have the highest metastatic potency, which may also offer a genetic explanation why rare skip metastases of the level IV or level V were found in HNSCC (Lydiatt et al., 2017).

The relatively small quantity of lymphatic metastasis samples, the variations of tumor sample purity, and the intratumor heterogeneity limit our current study, especially given that the expression of *SMAD3, PECAM1, TMEM173, STAT3, and TWISTNB* reveals no statistically significant difference among different groups. Meanwhile, our observation revealed *PECAM1* was significantly decreased but it showed no significant difference in TCGA datasets. It may result from that the squamous cell cancer in oropharynx, hypopharynx, and larynx were included in TCGA dataset, and they had an entirely different biological behavior and prognosis from HNSCC. While these findings will require further validation in larger cohorts of patient samples in the future, we believe this first gene expression analysis of cervical lymph nodes in the individual level of cancer patients will provide us an important opportunity to guide the future investigations of HNSCC.

Through gene expression analyses, we found that the mRNA expression of selected CSCs and immune suppression markers exhibit the highest expression in the level II metastatic lymph nodes, then declined in the level III and remained constant at a reduced value in levels IV and V metastatic lymph nodes. These results help to explain the reason why the level II has a high incidence of lymph node metastasis, and skip metastasis to the level IV or level V is rarely found in the clinic. It will help to increase the understanding of the genetic characteristics associated with metastatic loci and potential routes of lymphatic dissemination of HNSCC, and may aid the clinical diagnosis and treatment of HNSCC.

## Data Availability Statement

All datasets generated for this study are included in the article/Supplementary Material.

## Ethics Statement

The studies involving human participants were reviewed and approved by Shanghai Ninth People's Hospital Ethical Committee. The patients/participants provided their written informed consent to participate in this study.

## Author Contributions

SQ wrote the paper. ZH, RY and LL carried out experimental studies. LM and SL were involved in statistical analysis. GR and JH modified the paper and designed this study concepts. All authors read and approved the final manuscript.

### Conflict of Interest

The authors declare that the research was conducted in the absence of any commercial or financial relationships that could be construed as a potential conflict of interest.
