# Supplementary material for: Validation of Gene Profiles for Analysis of Regional Lymphatic Metastases in Head and Neck Squamous Cell Carcinoma
Source: Front Mol Biosci. 2020 Feb 4;7:3. doi: 10.3389/fmolb.2020.00003 (PMC7010860; doi:10.3389/fmolb.2020.00003)
Supplement: Supplementary file 1 [file Table_1.DOC]

**
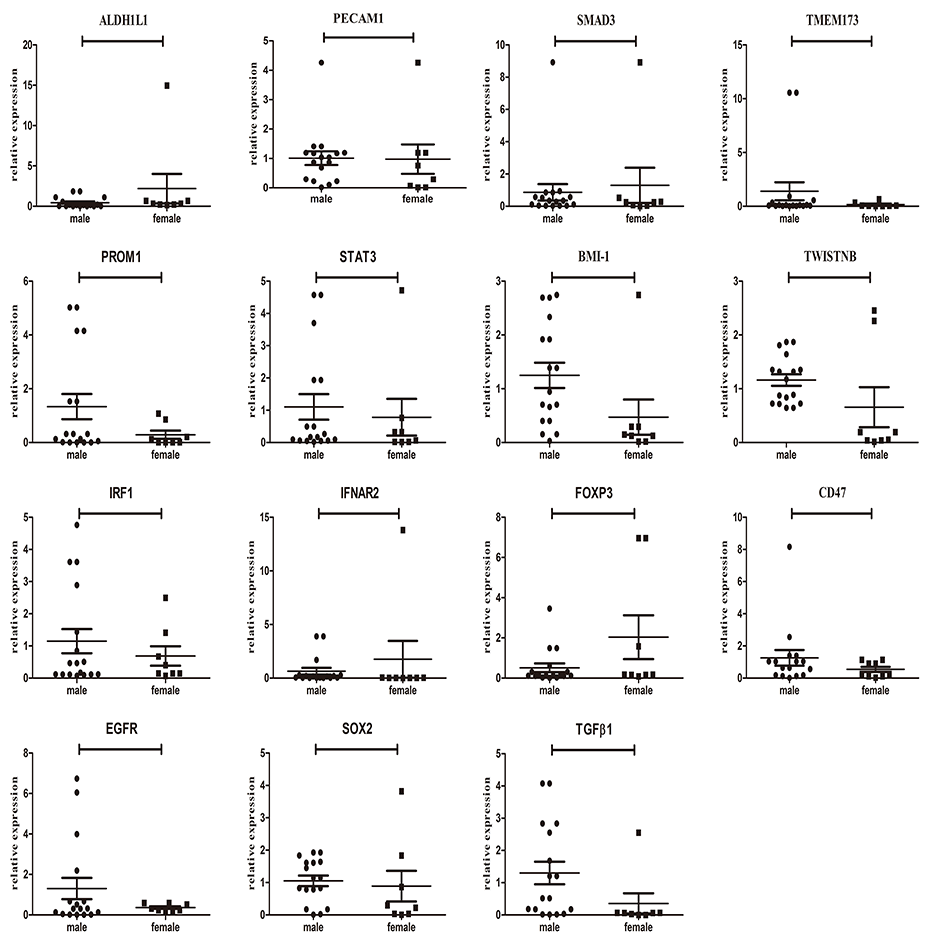
**

**Figure S1.** **mRNA expression of selected genes in adjacent normal tissue samples between male and female patients.**

**
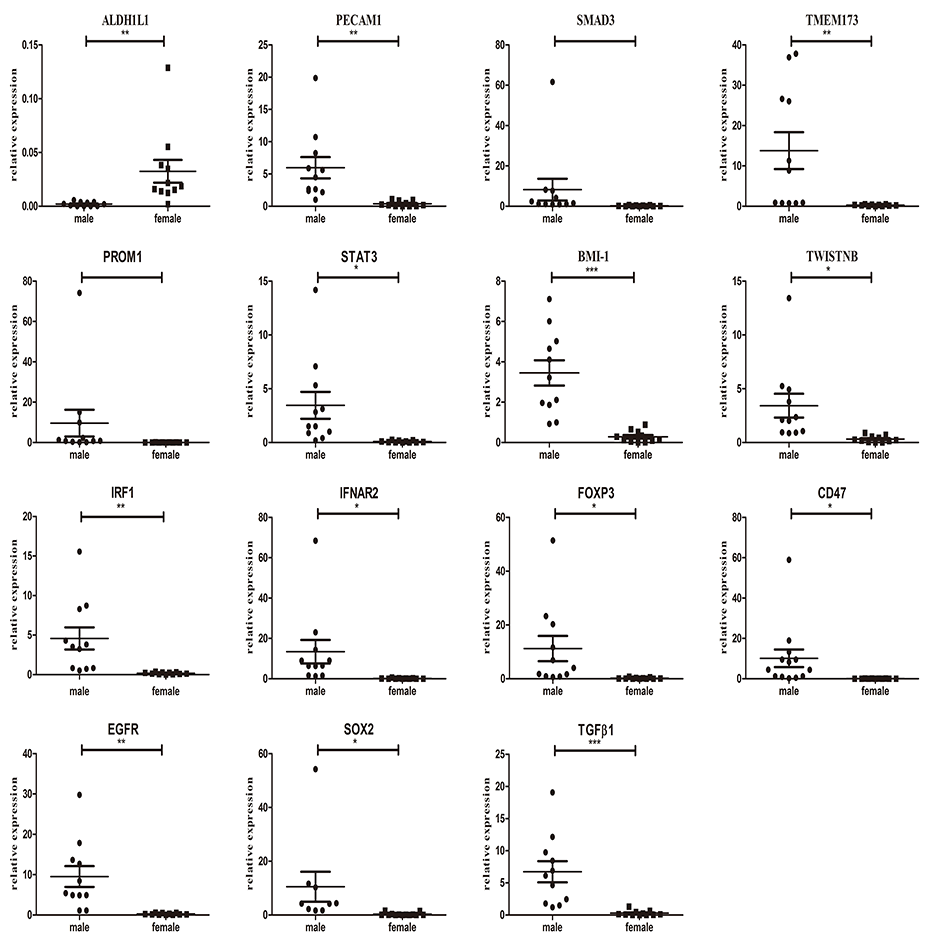
**

**Figure S2. mRNA expression of selected genes in the metastatic lymph nodes between male and female patients.**

**Table S1 the primer sequences of the selected 15 genes**

| gene | Forward Primer (5' -> 3') | Reverse Primer (5' -> 3') |
| --- | --- | --- |
| IRF1 | ATGCCCATCACTCGGATGC | CCCTGCTTTGTATCGGCCTG |
| IFNAR2 | TCATGGTGTATATCAGCCTCGT | AGTTGGTACAATGGAGTGGTTTT |
| FOXP3 | GTGGCCCGGATGTGAGAAG | GGAGCCCTTGTCGGATGATG |
| TMEM173 | CCAGAGCACACTCTCCGGTA | CGCATTTGGGAGGGAGTAGTA |
| CD47 | AGAAGGTGAAACGATCATCGAGC | CTCATCCATACCACCGGATCT |
| PECAM1 | AACAGTGTTGACATGAAGAGCC | TGTAAAACAGCACGTCATCCTT |
| BMI-1 | CCACCTGATGTGTGTGCTTTG | TTCAGTAGTGGTCTGGTCTTGT |
| TWISTNB | GCCTAGAGTTGCCGACTTATG | TGCGTTTCCTGTTAAGGTAGC |
| ALDH1L1 | AGATTGCAGTGATTGGACAGAG | CCAAAGCCTGGTATTTTGCCA |
| PROM1 | AGTCGGAAACTGGCAGATAGC | GGTAGTGTTGTACTGGGCCAAT |
| EGFR | AGGCACGAGTAACAAGCTCAC | ATGAGGACATAACCAGCCACC |
| SOX2 | GCCGAGTGGAAACTTTTGTCG | GGCAGCGTGTACTTATCCTTCT |
| TGFB1 | GGCCAGATCCTGTCCAAGC | GTGGGTTTCCACCATTAGCAC |
| SMAD3 | GCGTGCGGCTCTACTACATC | GCACATTCGGGTCAACTGGTA |
| STAT3 | CAGCAGCTTGACACACGGTA | AAACACCAAAGTGGCATGTGA |
